# Obesity Hypoventilation Syndrome-Related Challenges in Acute Respiratory Failure

**DOI:** 10.7759/cureus.18066

**Published:** 2021-09-17

**Authors:** Michael Dougherty, Christine M Lomiguen, Justin Chin, Philip K McElroy

**Affiliations:** 1 Department of Anesthesiology, Thomas Jefferson University Hospital, Philadelphia, USA; 2 Department of Medical Education, Lake Erie College of Osteopathic Medicine, Erie, USA; 3 Department of Family Medicine, Millcreek Community Hospital, Erie, USA; 4 Department of Family Medicine, LifeLong Medical Care, Richmond, USA

**Keywords:** morbidity, prolonged ventilation, obesity, morbid obesity, super super morbid obesity, hypoventilation, comorbid obesity, obesity hypoventilation syndrome, covid-19, ohs

## Abstract

Obesity hypoventilation syndrome (OHS) is a condition commonly found in severely obese patients in which they fail to breathe deeply or rapidly enough to offset the body’s need for oxygen consumption and carbon dioxide release. This report presents a case of a 49-year-old super-super-morbid obese female with a body mass index (BMI) of 90 kilogram per meter squared (kg/m²), chronic obstructive pulmonary disease (COPD), and end-stage cor pulmonale, who was brought to the emergency department for altered mental status and requiring emergent airway due to respiratory failure secondary to OHS. The continued increase in rates of obesity worldwide, especially in those with BMI ≥ 50 kg/m², may lead to an increase in the incidence of OHS. With comorbidities secondary to obesity and associated complexity, this medically challenging case emphasizes the need for refined management strategies in caring for OHS in super-super-morbidly obese patients.

## Introduction

Obesity hypoventilation syndrome (OHS) is a condition commonly found in severely obese patients in which they fail to breathe deeply or rapidly enough to offset the body’s need for oxygen consumption and carbon dioxide release [1]. Often found in conjunction with other medical comorbidities, OHS can result in acute hypercapnic respiratory failure, particularly when long-term ventilation is required. Since the 1960s, obesity-associated sequelae have steadily risen with the increasing rates of obesity across the population in the United States [2]. Attributed to the increasing availability of highly processed foods and ease of sedentary lifestyles, the early 2000s are considered the start of the obesity “epidemic” that has now reached global proportions. The World Health Organization has released obesity classifications based on the body mass index (BMI), which utilizes an individual’s height and weight to calculate a number that ranges from <18.5 kilograms per meter squared (kg/m²) as underweight to ≥40.0 kg/m² as class III/morbid obesity [3]. Due to the increasing prevalence of patients in class III obesity, researchers have since created unofficial subclassifications of super-morbid obesity (BMI ≥ 50.0 kg/m²) and super-super-morbid obesity (BMI ≥ 60.0 kg/m²), highlighting the unique challenges associated with obesity [4].

Here we present a case of a 49-year-old super-super-morbid obese female (BMI 90 kg/m²) with chronic obstructive pulmonary disease (COPD) and end-stage cor pulmonale, brought to the emergency department for altered mental status and requiring emergent airway due to respiratory failure secondary to OHS. With the increasing complexity of patients and comorbidities secondary to obesity, this medically challenging case emphasizes the need for refined management strategies in caring for OHS in super-super-morbidly obese patients.

## Case presentation

**Figure 1:**
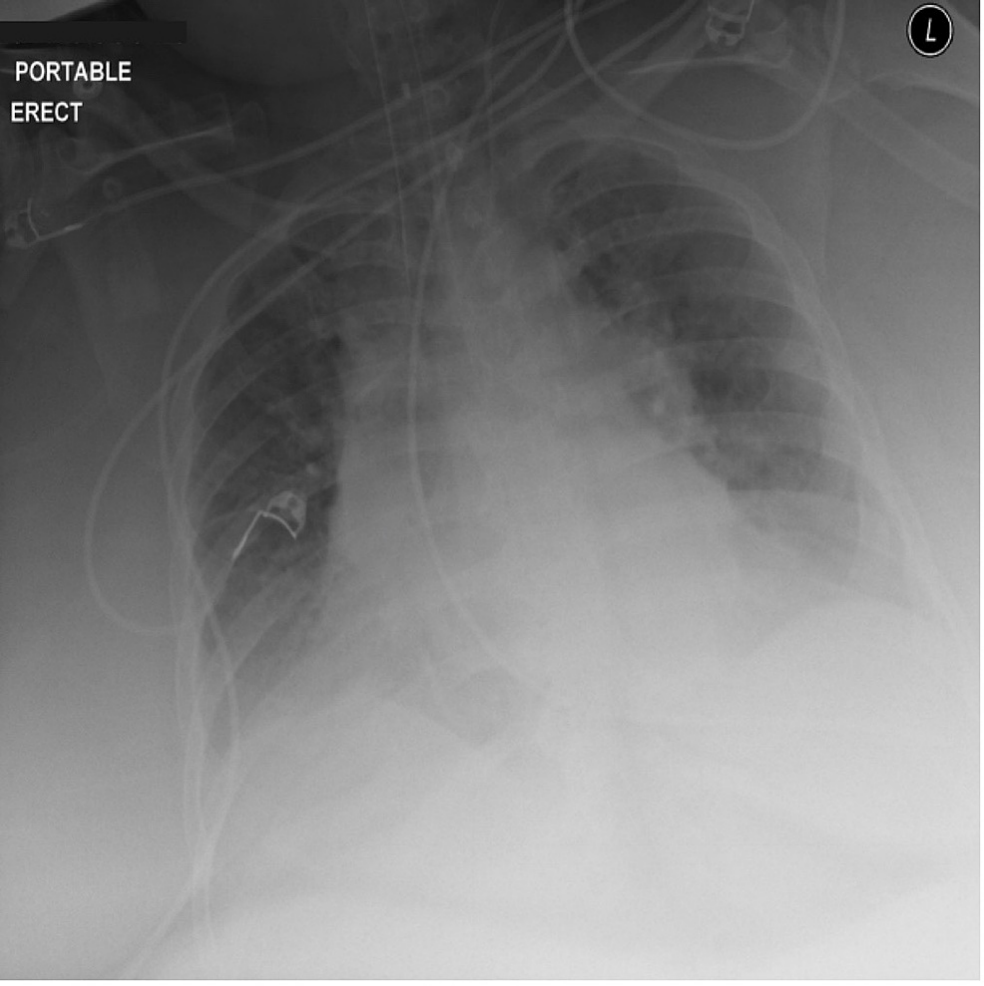
Portable anteroposterior chest X-ray showing hyperinflation of lungs bilaterally. Cardiac silhouette and other radiographic landmarks are difficult to appreciate secondary to body habitus.

A 49-year-old super-super-morbidly obese female (BMI 90 kg/m²) with a past medical history of chronic obstructive pulmonary disease and cor pulmonale presented to the emergency department with altered mental status and difficulty ambulating secondary to lower extremity edema. Vitals revealed an elevated heart rate of 100 beats per minute and decreased respiratory rate of 10 breaths per minute. Physical exam was significant for lethargic mentation and denuded abdominal wounds under her pannus. Bi-level positive airway pressure and intravenous clindamycin were started due to hypercapnic respiratory failure and concerns for infection. Chest X-ray showed hyperinflated lung volumes without obvious signs of pneumonia (Figure 1). Arterial blood gases demonstrated elevated serum bicarbonate, consistent with chronic carbon dioxide retention. The patient was admitted to the intensive care unit and emergently intubated due to worsening respiratory status. General surgery recommended discontinuation of clindamycin in favor of local wound care.

On hospitalization day seven, palliative care was consulted around goals of care and tracheostomy tube placement. Percutaneous endoscopic gastrostomy (PEG) tube or gastrostomy tube placement were discussed for nutrition; however, the ICU and surgical teams presented concerns of abdominal wall thickness and potential displacement into the peritoneal and abdominal cavity. The family was counseled regarding the risks and benefits and elected to proceed with a tracheostomy tube and PEG placement with full code status. Standard approaches for tracheostomy and PEG tube placement were performed. Directly post-procedure, however, the patient developed atrial fibrillation with a rapid ventricular response. Advanced cardiac life support (ACLS) protocols with phenylephrine, midazolam, cardioversion, and amiodarone were successively given in addition to a heparin drip, which stabilized the patient. Two hours after direct current cardioversion, the patient became hypotensive, and an arterial line was placed. After arterial line placement, the patient returned to atrial fibrillation and hypotension.

Following substantial worsening in the patient’s clinical picture and vitals (oxygenation levels dropping below 86% and difficulty breathing), the critical and intensive care teams increased the positive end-expiratory pressure (PEEP) and tidal ventilation (VT) secondary to the patient's body habitus. During these attempts to stabilize the patient, it was observed that the balloon on the Shiley tracheostomy was deflated and subsequent attempts to inflate were unsuccessful. The patient was removed from the ventilator while manual ventilation took place and subsequently intubated orotracheally. Although the patient was able to be ventilated manually to 100% oxygen saturation after intubation and decannulation, however, would spontaneously desaturate despite increased oxygenation levels, ventilation rates, and vecuronium. Cardiopulmonary resuscitation (CPR) was initiated per ACLS protocol, in which return of spontaneous circulation (ROSC) was achieved for one minute, however, quickly became pulseless again.

The patient's family was notified during the start of the code and arrived during ongoing CPR. The family asked to stop CPR after 12 rounds of epinephrine. CPR was discontinued and the patient was asystolic.

## Discussion

Many countries are now experiencing excess weight and obesity as an epidemic. The prevalence of obesity among adults in the United States has steadily been increasing in the past two decades and is projected to exponentially grow in the next decade [5]. Obesity creates increasing complexity in healthcare as it insidiously affects all organs systems. The pulmonary system is not spared as studies have shown to cause decreased lung volumes, increased respiratory resistance, and decreased chest wall compliance [6,7]. While obesity is frequently observed in conjunction with COPD, asthma, and sleep-apnea-hypopnea syndrome, only a minority of these patients develop obesity hypoventilation syndrome. As diagnoses of obesity increase, so do the prevalence of OHS, with an estimation of 0.3%-0.4% in the general population, 10%-20% in patients with sleep-related breathing disorders, and close to 50% of those hospitalized with BMI greater than 50 kg/m² [8,9].

As defined by the American Thoracic Society, OHS is the triad of obesity (BMI ≥ 30 kg/m2), awake hypoventilation with partial pressure of carbon dioxide (PaCO2) ≥ 45 millimeter of mercury (mmHg), and with sleep-disordered breathing in the absence of a neuromuscular, mechanical, or metabolic explanation for hypoventilation [1]. Management of OHS, especially in the context of acute hypercapnic respiratory failure, is challenging as traditional therapies used to treat respiratory failure may have adverse secondary outcomes. For example, oxygen administration is often first-line in respiratory failure; however, in patients with OHS, excessive administration of oxygen may lead to acute hyperoxia-induced hypercapnia and reduced minute ventilation [10,11]. The reversal of hypoxic pulmonary vasoconstriction leads to greater blood flow to poorly ventilated segments, in which close titration of supplemental oxygen has been shown to have improved outcomes. Lower extremity edema is also common in OHS patients due to cor pulmonale [12]. As evidenced in this patient’s case, left ventricular diastolic dysfunction is associated with her super-super-morbid obesity, which was likely secondary to hypertension or obesity cardiomyopathy, or other comorbidities. As such, these patients are typically treated aggressively with a loop diuretic; however, it has been shown that diuresis prior to the resolution of the increased vascular resistance can result in prerenal or cardiorenal azotemia [13]. This did not occur with the case patient and although this phenomenon has not been well studied in patients with OHS, similar observations have been made in those with COPD [11,12].

Although the exact mechanisms of hypercapnia in OHS are not well understood, patients may have altered reactivity to carbon dioxide. In addition to hypercapnia, OHS has been associated with metabolic alkalosis, hypoxemia, and hypoventilation, all of which drive additional right heart failure and lower extremity edema, thus requiring additional diuresis [1,2]. With OHS, total respiratory compliance can be altered by up to as much as two-thirds of normal values due to fat distribution in and around the ribs, diaphragm, and abdomen [14]. The effect of obesity on lung volumes has even worse implications during anesthesia and muscle paralysis, especially with abdominal surgery [15]. In prior studies, the differences of maximum inspiratory and expiratory pressures at all lung volumes between the obese and normal subjects are lower in the obese but do not reach statistical significance unless the patient has OHS [15,16]. The maximum voluntary ventilation is reduced by 20% in healthy obese individuals and by 45% in those with OHS, which is important in light of the ongoing coronavirus disease 2019 (COVID-19) pandemic as it can complicate oxygenation and life-saving measures such as intubation [17].

## Conclusions

OHS is a poorly understood etiology of hypercapnic respiratory failure. Continued increases in rates of obesity, especially in those with BMI ≥ 50 kg/m², may lead to an increase in the incidence of OHS. Understanding the underlying pathophysiology of the condition in addition to the stress obesity places on the respiratory system may lead to improved treatment of these patients. In the setting of a respiratory emergency, standards of care of respiratory distress may lead to worse outcomes. It is plausible that a more nuanced approach to respiratory failure in patients with OHS can improve mortality.
